# Multi-omics analyses unravel DNA damage repair-related clusters in breast cancer with experimental validation

**DOI:** 10.3389/fimmu.2023.1297180

**Published:** 2023-10-31

**Authors:** Peng Liu, Xinpei Deng, Huamao Zhou, Jindong Xie, Yanan Kong, Yutian Zou, Anli Yang, Xing Li

**Affiliations:** ^1^ State Key Laboratory of Oncology in South China, Guangdong Provincial Clinical Research Center for Cancer, Sun Yat-sen University Cancer Center, Guangzhou, China; ^2^ The Affiliated Nanhua Hospital, Hengyang Medical School, University of South China, Hengyang, China

**Keywords:** breast cancer, DNA damage repair, machine learning, single-cell, PRAME

## Abstract

**Background:**

As one of the most common malignancies worldwide, breast cancer (BC) exhibits high heterogeneity of molecular phenotypes. The evolving view regarding DNA damage repair (DDR) is that it is context-specific and heterogeneous, but its role in BC remains unclear.

**Methods:**

Multi-dimensional data of transcriptomics, genomics, and single-cell transcriptome profiling were obtained to characterize the DDR-related features of BC. We collected 276 DDR-related genes based on the Molecular Signature Database (MSigDB) database and previous studies. We acquired public datasets included the SCAN-B dataset (GEO: GSE96058), METABRIC database, and TCGA-BRCA database. Corresponding repositories such as transcriptomics, genomics, and clinical information were also downloaded. We selected scRNA-seq data from GEO: GSE176078, GSE114727, GSE161529, and GSE158724. Bulk RNA-seq data from GEO: GSE176078, GSE18728, GSE5462, GSE20181, and GSE130788 were extracted for independent analyses.

**Results:**

The DDR classification was constructed in the SCAN-B dataset (GEO: GSE96058) and METABRIC database, Among BC patients, there were two clusters with distinct clinical and molecular characteristics: the DDR-suppressed cluster and the DDR-active cluster. A superior survival rate is found for tumors in the DDR-suppressed cluster, while those with the DDR-activated cluster tend to have inferior prognoses and clinically aggressive behavior. The DDR classification was validated in the TCGA-BRCA cohort and shown similar results. We also found that two clusters have different pathway activities at the genomic level. Based on the intersection of the different expressed genes among these cohorts, we found that PRAME might play a vital role in DDR. The DDR classification was then enabled by establishing a DDR score, which was verified through multilayer cohort analysis. Furthermore, our results revealed that malignant cells contributed more to the DDR score at the single-cell level than nonmalignant cells. Particularly, immune cells with immunosuppressive properties (such as FOXP3+ CD4+ T cells) displayed higher DDR scores among those with distinguishable characteristics.

**Conclusion:**

Collectively, this study performs general analyses of DDR heterogeneity in BC and provides insight into the understanding of individualized molecular and clinicopathological mechanisms underlying unique DDR profiles.

## Introduction

As one of the most common malignancies worldwide, breast cancer (BC) exhibits high heterogeneity of molecular phenotypes (1). According to the status of the human epidermal growth factor receptor-2 (HER2) and hormone receptor (HR), BC can be divided into four subgroups: luminal A (HR+/HER2-), luminal B (HR+/HER2+), HER2-positive (HR-/HER2+), and triple-negative breast cancer (HR-/HER2- [TNBC]) (2). BC is usually associated with unfavorable survival rates and requires molecules that assist in determining prognoses as well as monitoring efficacy. There have been significant advances in understanding the molecular heterogeneity of BC over the past few decades (3–5). These studies provide comprehensive insights into the molecular phenotypes of BC and offer effective options. Nevertheless, molecular mechanisms behind the dismal prognosis of BC remain unclear. Therefore, it is critical to recognize the molecular characteristics and mechanisms of BC.

Genome stability depends on DNA damage repair (DDR). It is well known that DDR pathways involve in the onset, progression, and therapeutic response of tumor (6). Besides, treatment strategies that target DDR are increasingly coming to fruition. Poly (ADP-ribose) polymerase (PARP) is a classic enzyme that detects DNA damage and has been applied as a promising target (7). Based on genetic, biochemical, and mechanistic criteria, DDR-related genes can be categorized into several pathways (8). Numerous studies have provided insights into the mechanism and therapeutic efficacy of DDR in multiple cancer types (9–12). However, most of them focused on the bulk transcriptome analysis, and multi-dimensional analysis is still lacking, especially in BC. The BC ecosystem consists of diverse molecular characteristics and immune infiltrations, and DDR zealously contributes to the processes of BC carcinogenesis as well as immune characteristics. Recently, Ka et al. identified that IFI16 could inhibit DDR that potentiates type-I interferon-induced antitumor effects in TNBC (13). However, these studies predominantly concentrated on single gene. Besides, single-cell RNA sequencing (scRNA-seq) technology offers an accurate way to identify both intrinsic and extrinsic characteristics of tumor cells (14, 15). It is capable of identifying different cell subsets, illustrating clonal diversity, and, importantly, figuring out the critical factor influencing tumor heterogeneity (16). By identifying cancer subtypes, patients can be tracked for treatment responses and improvement in outcomes (17–21). Consequently, uncovering the roles of DDR in BC is imperative.

Herein, we performed a multi-dimensional analysis of transcriptomics, genomics, and single-cell transcriptome profiling to characterize the DDR-related features of BC. We defined DDR-related clusters based on 276 DDR-related genes. The DDR classification was then enabled by establishing a DDR score, which was verified through multilayer cohort analysis. Furthermore, we distinguished DDR characteristics between nonmalignant and malignant cells, as well as among immune cell subtypes. Collectively, our findings provided general analyses of DDR heterogeneity in BC which might help with prognosis monitoring and diverse therapies.

## Materials and methods

### Patients and samples

We acquired public datasets included the SCAN-B dataset (GEO: GSE96058) (22), METABRIC database (Dataset ID: EGAS00001001753) (23), and TCGA-BRCA database. Corresponding repositories such as transcriptomics, genomics, and clinical information were also downloaded. We selected scRNA-seq data from GEO: GSE176078 (24), GSE114727 (25), GSE161529 (26), and GSE158724 (27). Bulk RNA-seq data from GEO: GSE176078 (24), GSE18728 (28), GSE5462 (29), GSE20181 (30), and GSE130788 (31) were extracted for independent analysis. The probes were mapped using “AnnoProbe” R package. “GeoTcgaData” R package was applied to convert ensemble ids to gene symbols. The average values of multiple probes were calculated by the “limma” R package if necessary.

### Construction and validation of the DDR-related clusters

To identify potential subtypes of breast cancer, we searched for 276 DDR-related genes based on the Molecular Signature Database (MSigDB) database and previous studies (8). These DDR genes involved in ten DDR pathways. Detailed information was summarized in **Supplementary T** **able S1**. We then performed the ssGSEA algorithm (“GSVA” R package) to calculate the enrichment level of each DDR pathway as well as total DDR score in each sample through transcriptomics.

We applied “ConsensusClusterPlus” R package to determine the optimal k value for the DDR-related clusters (32). The CC parameter “maxK” was selected as “8”, “clusterAlg” was selected as “km”, and “distance” was selected as “pearson”. Heatmaps were shown with “ComplexHeatmap” R package (33). PCA was also used to show the heterogeneity of the clusters.

### Survival analysis

K-M analyses of OS, BCSS, and DFS were performed by “survival” and “survminer” R packages and shown in “ggsurvplot” R package. The median value was used as the cut-off value.

### Collection and calculation of signature scores

We collected 50 hallmark signatures retrieved from MSigDB. ssGSEA algorithm (“GSVA” R package) was applied to assess the enrichment level of each hallmark signature in each sample (34). 31 COSMIC signatures were obtained from online website (https://software.broadinstitute.org/cancer/cga/msp). The COSMIC signature scores were calculated by “deconstructSigs” R package (35).

### DEGs analysis

We conducted DEGs analysis by the “limma” R package for the SCAN-B and METABRIC cohorts, and the “edgeR” R package for the TCGA-BRCA cohort (36, 37). Genes with *adjusted P* < 0.05 and an absolute log2 fold change (FC) > 0.5 were considered as DEGs. Common DEGs were shown by the “ggvenn” R package.

### Human BC cell lines and cell culture

We used the human epithelial BC cell lines, including MCF10A, T47D, MCF7, ZR-75-1, SKBR-3, and MDA-MB-468. The cell lines present in this study were obtained from the American Type Culture Collection. Standard guidelines were followed to culture all cell lines and maintain them at 37°C and 99% relative humidity without antibiotics.

### RNA isolation and qRT-PCR analysis

We extracted total RNA from cells using the RNA-Quick Purification Kit (ES-RN001, Shanghai Yishan Biotechnology Co., Shanghai, China). **Supplementary T** **able S2** showed the primer sequences. We used qRT-PCR with three technical repetitions to determine RNA levels on a Bio-Rad CFX96 using the SYBR Green method (RR420A, Takara, Mountain View, CA, USA). We purchased the qRT-PCR plate from NEST (402301, Wuxi NEST Biotechnology Co., Jiangsu, China). Comparative Ct method was used to normalize RNA levels against β-actin RNA.

### Western blot analysis

Protein extracts from cells were prepared using RIPA lysis buffer. Total protein was added to SDS-PAGE and transferred to PVDF membrane (Millipore). Antibody against PRAME and GAPDH was used. Membrane was incubated with primary antibody at 4°C overnight and subsequent secondary antibody at room temperature for 1h. The blots were further visualized with Immobilon Western Chemiluminescent HRP Substrate (Beyotime).

### scRNA-seq data processing

Annotated cell types of the GEO: GSE176078 and GSE114727 datasets, and the clinical information of the GEO: GSE161529 and GSE158724 datasets were all obtained from their previous studies. “Seurat” R package was applied to accomplish subsequent analysis (38). We selected the top 2000 highly variable genes (HGVs). The process involved the utilization of the top 20 principal components, in conjunction with HGVs. We used t-SNE to reduce the dimensions and observe the classification of each cell type. According to previously reported markers, CD4+ T cells, myeloid cells, CD8+ T cells, and macrophages were furtherly distinguished (5, 39).

### Statistical analysis

We used R version 4.1 to perform all analyses. We used Wilcoxon rank-sum test to compare the differences between two groups and Kruskal-Wallis test to three or more groups. The hazard ratios (HR) and 95% confidence intervals (CI) were also calculated.

Chisquare test was applied for two groups of categorical variables. We chose pearson correlation coefficient to accomplish correlation analysis. *P* < 0.05 was considered statistically significant.

## Result

### BC has two types of statuses based on the DDR-related genes

**Figure 1:**
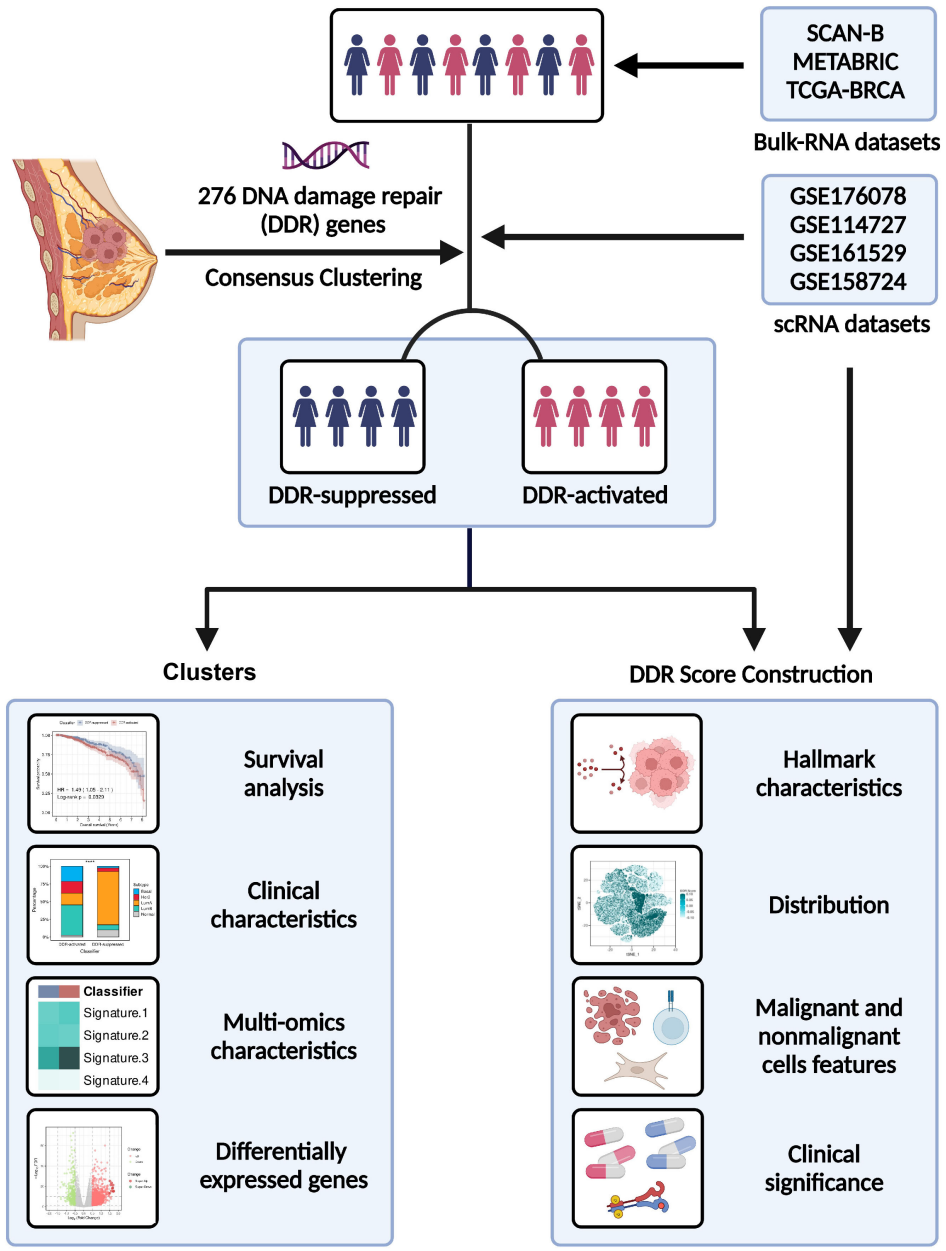
Flowchart of our study.

**Figure 2:**
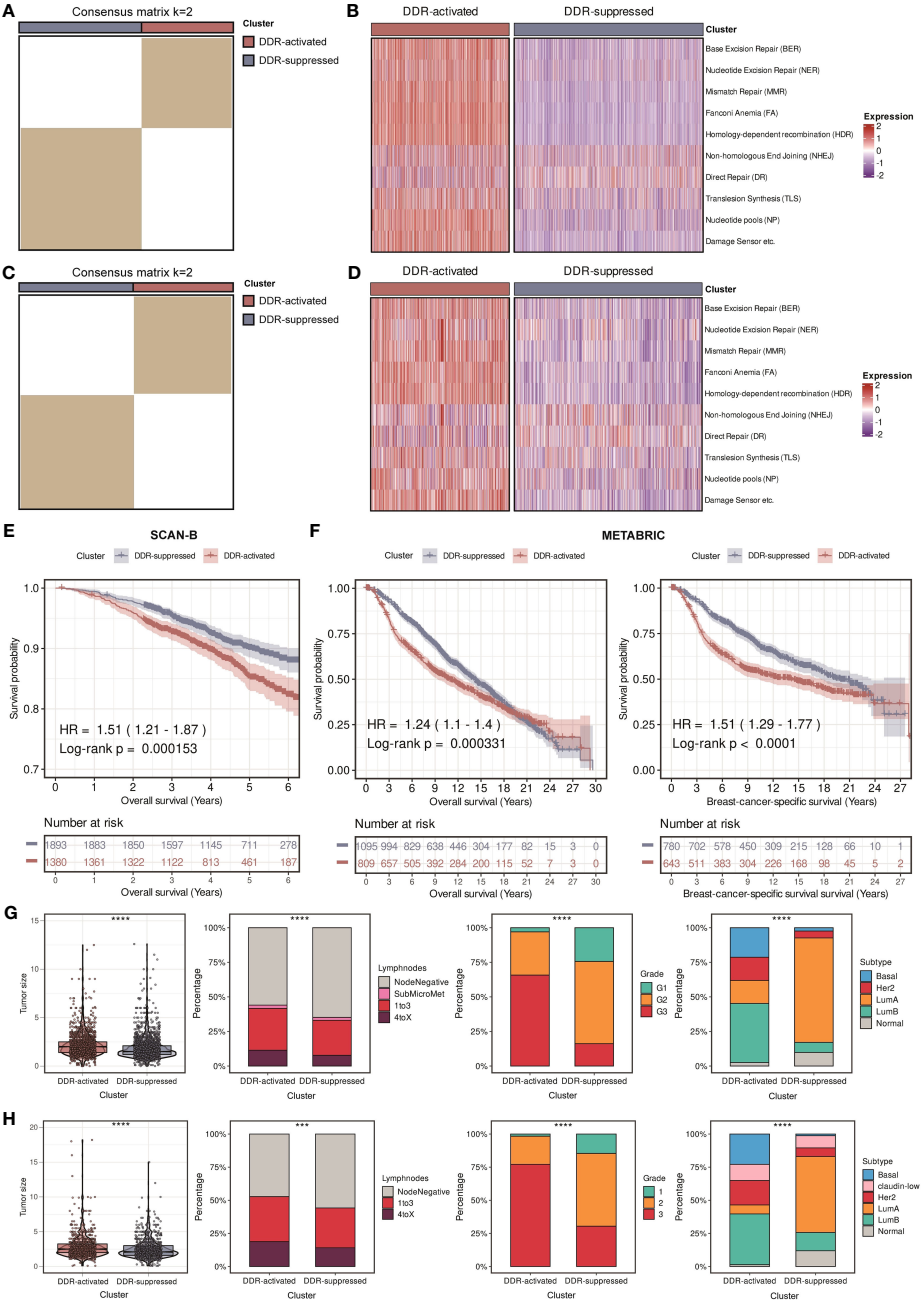
Identification of two DDR-related clusters in the SCAN-B and METABRIC cohorts. **(A)** Heatmap displaying consensus clustering with the robust classification in the SCAN-B cohort (k = 2). **(B)** Heatmap based on ten DDR pathways calculated through ssGSEA algorithm in the SCAN-B cohort. **(C)** Heatmap displaying consensus clustering with the robust classification in the METABRIC cohort (k = 2). **(D)** Heatmap based on ten DDR pathways calculated through ssGSEA algorithm in the METABRIC cohort. **(E)** Kaplan-Meier curves of OS between two DDR-related clusters in the SCAN-B cohort. **(F)** Kaplan-Meier curves of OS and BCSS between two DDR-related clusters in the METABRIC cohort. **(G)** Violin plots and bar plots of clinical features (tumor size, positive lymphnodes, pathological grade, and PAM50 subtypes) between two DDR-related clusters in the SCAN-B cohort. **** means *P* < 0.0001. **(H)** Violin plots and bar plots of clinical features (tumor size, positive lymphnodes, pathological grade, and PAM50 subtypes) between two DDR-related clusters in the METABRIC cohort. **** means *P* < 0.0001 and *** means *P* < 0.001.

In order to provide a systematic description of our study, a flowchart was developed (**Figure 1**). We accomplished unsupervised clustering in the SCAN-B and Molecular Taxonomy of Breast Cancer International Consortium (METABRIC) dataset based on 276 DDR-related genes. We found that when k = 2, two subgroups of patients could be distinguished in each cohort (**Figures 2A, C**). Using the single-sample gene set enrichment analysis (ssGSEA) algorithm, we estimated the enrichment level of ten DDR-related pathways in each sample. We found that two clusters had distinct DDR characteristics (**Figures 2B, D**). The DDR-suppressed subgroup was determined by the relative downregulation of most DDR-related pathways, which was contrary to that in the DDR-activated subgroup. Additionally, overall survival (OS) outcomes were evidently different between the subgroups in the SCAN-B cohort, with DDR-activated subgroup showing inferior OS (**Figure 2E**). Similar result was then confirmed in the METABRIC cohort. The DDR-activated subgroup was related to poorer breast-cancer-specific survival (BCSS) and OS (**Figure 2F**). Furthermore, we compared clinical characteristics in the two cohorts. **Figures 2G**, **H** showed that the DDR-activated subgroup had larger tumor size (both *P* < 0.0001), higher possibility of lymph node metastasis (*P* < 0.0001 in the SCAN-B cohort; *P* < 0.001 in the METABRIC cohort), advanced grade (both *P* < 0.0001), and higher frequency of basal-like PAM50 subtype (both *P* < 0.0001).

### Further validation using the DDR clusters

**Figure 3:**
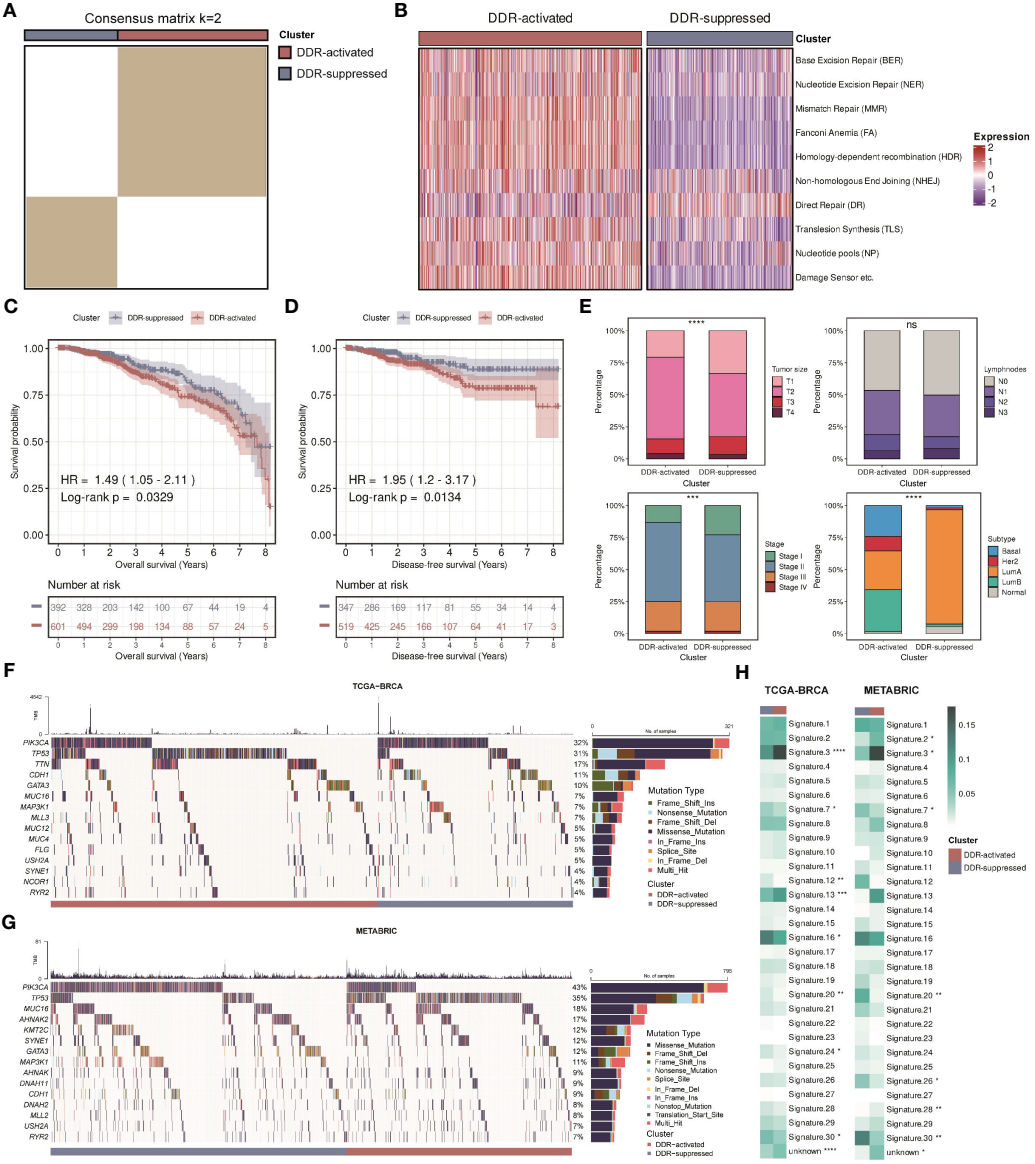
Validation of two DDR-related clusters in the TCGA cohort. **(A)** Heatmap displaying consensus clustering with the robust classification in the TCGA cohort (k = 2). **(B)** Heatmap based on ten DDR pathways calculated through ssGSEA algorithm in the TCGA cohort. **(C)** Kaplan-Meier curves of OS between two DDR-related clusters in the TCGA cohort. **(D)** Kaplan-Meier curves of DFS between two DDR-related clusters in the TCGA cohort. **(E)** Bar plots of clinical features (tumor size, positive lymphnodes, pathological stage, and PAM50 subtypes) between two DDR-related classifiers in the TCGA cohort. **** means *P* < 0.0001, *** means *P* < 0.001, and ns means no significance. **(F)** Mutation landscape between two DDR-related clusters in the TCGA cohort. **(G)** Mutation landscape between two DDR-related clusters in the METABRIC cohort. **(H)** Heatmaps of COSMIC signatures between two DDR-related clusters in the TCGA and METABRIC cohort. ****, ***, **, * means *P* < 0.0001, *P* < 0.001, *P* < 0.01, and *P* < 0.05, respectively.

The clusters were then validated in another large-scale cohort, The Cancer Genome Atlas (TCGA)-breast invasive carcinoma (BRCA) cohort. Based on the 276 DDR-related genes, a total of two subgroups were also identified in BC patients, the DDR-activated and DDR-suppressed subgroups (**Figure 3A**). **Figure 3B** revealed that similar changes in DDR-related pathways were found. Survival differences were also observed between two subgroups, that is, the DDR-activated subgroup was associated with worse OS and disease-free survival (DFS) (**Figures 3C, D**). Besides, we observed a similar relationship between the clusters and clinical parameters (**Figure 3E**). The DDR-activated subgroup is more likely to have larger tumor size (*P* < 0.0001), higher stage (both *P* < 0.001), and higher frequency of basal-like PAM50 subtype (*P* < 0.0001). Ultimately, the robustness of our DDR-related clusters was impressive, and it was able to establish two subgroups.

### DDR clusters-specific transcriptomic and genomic characteristics of BC

Previous studies have found that DDR can be triggered by amplifications or mutations in oncogenes (6, 40, 41). Hence, we examined gene mutation differences between two DDR subgroups in the TCGA-BRCA and METABRIC cohorts. We found that there was a high incidence of mutations in PIK3CA and TP53 in two cohorts of patients (**Figures 3F, G**). Since both genes are essential, our comparisons revealed that the DDR-activated subgroup had higher mutation rates of TP53, whereas the DDR-suppressed subgroup had higher mutation rates of PIK3CA. Moreover, we calculated 31 COSMIC signature scores related to the mutation characteristics. The values refer to the average contribution across all samples within each cluster. Previous studies indicated that “Signature 3”, “Signature 4”, “Signature 6”, “Signature 15”, “Signature 20”, and “Signature 26” were strongly correlated with DDR activity (35). “Signature 3” is mainly related to homology-dependent recombination (HDR), while “Signature 4”, “Signature 6”, “Signature 15”, “Signature 20”, and “Signature 26” are relevant to defective DNA mismatch repair (MMR). **Figure 3H** showed that the DDR-activated subgroup had higher score of “Signature 3” while the DDR- suppressed subgroup had higher scores of “Signature 20” and “Signature 26”, demonstrating that two subgroups have different pathway activities at the genomic level.

**Figure 4:**
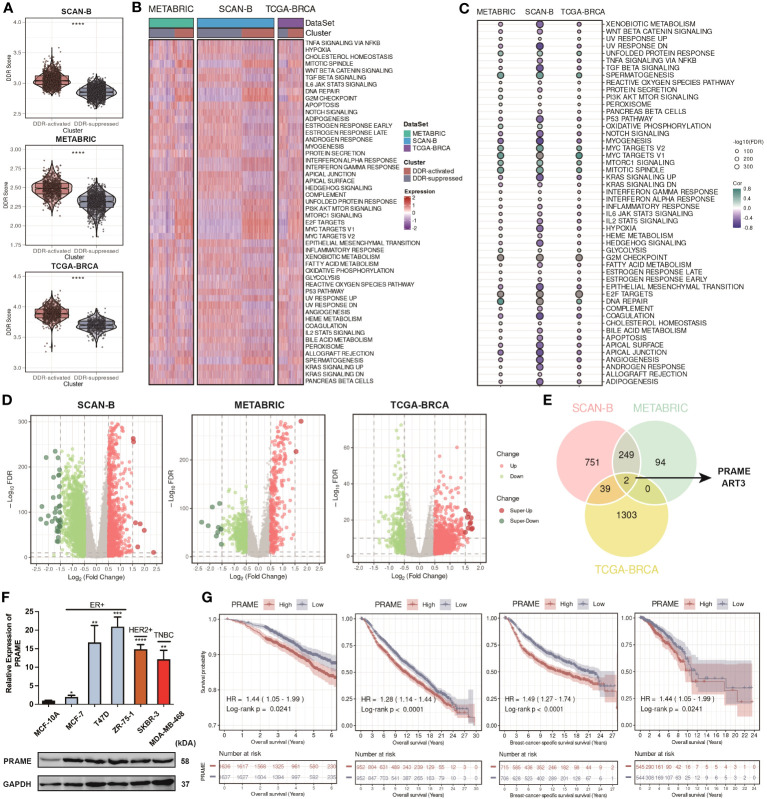
Construction of the DDR score and distinct multi-omics features of the DDR-related clusters revealing potential targets. **(A)** Violin plots of DDR score between two DDR-related clusters in the SCAN-B, METABRIC, and TCGA cohorts. **** means *P* < 0.0001. **(B)** Heatmap based on hallmark signatures calculated through ssGSEA algorithm in the SCAN-B, METABRIC, and TCGA cohorts. **(C)** Bubble plots of the correlation between DDR score and hallmark signatures in the SCAN-B, METABRIC, and TCGA cohorts. **(D)** Volcano plots of the DEGs between two DDR-related clusters in the SCAN-B, METABRIC, and TCGA cohorts. **(E)** Venn plot of the common DEGs among the SCAN-B, METABRIC, and TCGA cohorts. **(F)** Boxplot of PRAME expression among different breast cancer cell lines by qRT-PCR, and Western blot analysis of PRAME. ****, ***, **, * means *P* < 0.0001, *P* < 0.001, *P* < 0.01, and *P* < 0.05, respectively. **(G)** Kaplan-Meier curves of OS and BCSS based on the median expression of PRAME in the SCAN-B, METABRIC, and TCGA cohorts.

Using the “ssGSEA” algorithm, we then calculated a novel index named DDR score in each patient. We compared the DDR score between two subgroups and found that it was significantly higher in the DDR-activated subgroup (**Figure 4A**). Besides, we collected 50 hallmark signatures and calculated the enrichment level of each hallmark signature in each sample. The result of the heatmap showed that the DDR-activated subgroup displayed higher activity of mitotic spindle, DNA repair, unfolded protein response, G2M checkpoint, interferon responses, PI3K-AKT-mTOR signaling, whereas the DDR- suppressed subgroup exhibited higher activity of TGF-beta signaling, apoptosis, WNT-beta-catenin signaling, etc. (**Figure 4B**). The correlation between the DDR score and each hallmark signature level was also analyzed, and consistent conclusion was found (**Figure 4C**). Moreover, we constructed volcano plots to test whether the DDR-activated subgroup has uniquely expressed gene programs that might support their DDR status (**Figure 4D**). Based on the intersection of the upregulated genes among these cohorts, PRAME and ART3 appeared to be the most likely candidates (**Figure 4E**). Preferentially expressed antigen in melanoma (PRAME) is crucial for multiple cellular processes as well as immunotherapy response in human cancers among the cancer/testis antigen gene family (42). However, there have been no reports regarding the relationship between PRAME and DDR. We then applied quantitative real-time PCR (qRT-PCR) and Western blot analysis on PRAME. The results showed that that PRAME was significantly upregulated in multiple BC cell lines at both mRNA and protein levels (**Figure 4F**). Bioinformatic analysis in the TCGA-BRCA cohort also confirmed that PRAME was upregulated in tumor tissues compare with normal tissues (**Supplementary Figure S1**). Besides, our survival analysis among three cohorts displayed that PRAME was a significant risk factor for patients with BC (**Figure 4G**). Our findings indicated that PRAME might play a vital role in DDR.

### Malignant cells primarily contribute to DDR heterogeneity

**Figure 5:**
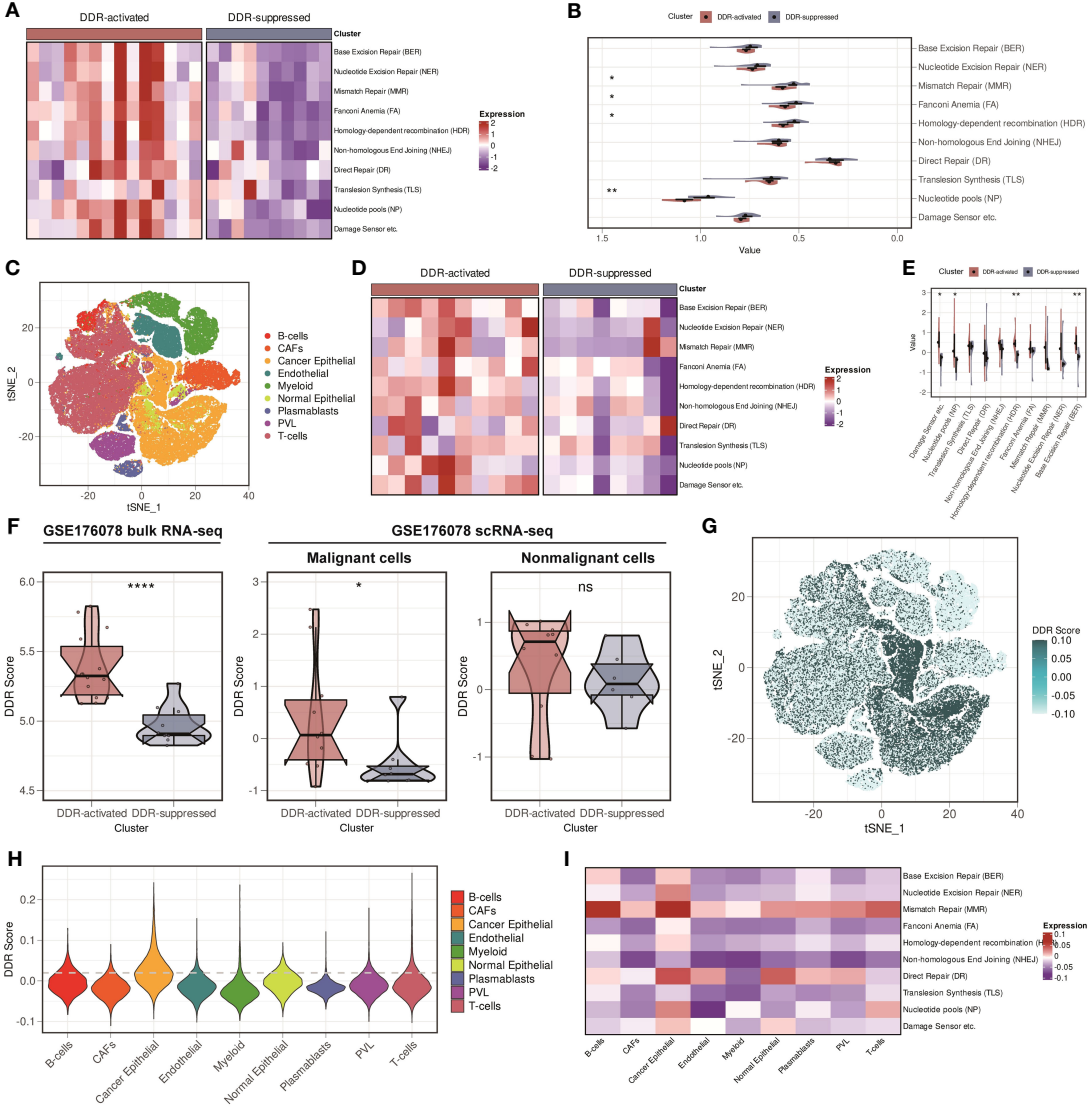
Validation of two DDR-related clusters in GSE176078, and malignant cells are the major contributor to DDR heterogeneity in breast cancer. **(A)** Heatmap based on ten DDR pathways calculated through ssGSEA algorithm in the GSE176078 bulk RNA-seq cohort. **(B)** Violin plots based on ten DDR pathways calculated through ssGSEA algorithm in the GSE176078 bulk RNA-seq cohort. ** means *P* < 0.01 and * means *P* < 0.05. **(C)** t-distributed stochastic neighbor embedding (t-SNE) plot of different cell types from GSE176078 scRNA-seq cohort. **(D)** Heatmap based on ten DDR pathways calculated through ssGSEA algorithm in the GSE176078 scRNA-seq cohort. **(E)** Violin plots based on ten DDR pathways calculated through ssGSEA algorithm in the GSE176078 scRNA-seq cohort. ** means *P* < 0.01 and * means *P* < 0.05. **(F)** DDR score among bulk RNA-seq (left), malignant cells (middle), and nonmalignant cells (right) between two DDR-related classifiers in the GSE176078 cohort. **** means P < 0.0001, * means P < 0.05 and ns means no significance. **(G)** t-SNE plot of the distribution of the DDR score in the GSE176078 scRNA-seq cohort. **(H)** Violin plot of the distribution of the DDR score in the GSE176078 scRNA-seq cohort. **(I)** Heatmap based on ten DDR pathways calculated through ssGSEA algorithm among different cell types in the GSE176078 scRNA-seq cohort.

Firstly, we verified our findings in another bulk RNA-seq cohort, GSE176078. We found that patients were stably classified into two subgroups, and **Figures 5A, B** showed that similar DDR-related pathways alteration was observed in GSE176078. Afterward, we investigated whether the difference of DDR status between the two subgroups can be determined at the single-cell level. By applying scRNA-seq technique, we can better understand the tumor diversity as well as tumor microenvironment (TME). We then downloaded the scRNA-seq data from GSE176078. Cells were broadly divided into 9 types, including B cells, cancer-associated fibroblasts (CAFs), cancer epithelial cells, endothelial cells, myeloid cells, normal epithelial cells, plasma blasts, perivascular-like cells (PVLs), and T cells (**Figure 5C**). Additionally, we stratified the samples by mixing the cells from each patient. Intriguingly, all patients were still allocated into two subgroups at the single-cell level, with similar conclusion found in the bulk RNA-seq level (**Figures 5D, E**). We subsequently assessed the DDR score of each patient. We found that the customized DDR score was significantly higher in the DDR-activated subgroup (*P* < 0.0001, **Figure 5F**). Besides, we evaluated the DDR score in each cell at the single-cell level. We found that the DDR score in malignant cells showed significant difference between two subgroups. Comparatively, the DDR score in nonmalignant cells did not differ significantly from those in the subgroup (**Figure 5F**). Besides, we explored the distribution of the DDR score among 9 cell types. The result showed that malignant cells exhibited the highest DDR activity (**Figures 5G, H**), which was accompanied by an upregulation of most DDR-related pathways (**Figure 5I**). In conclusion, malignant cells primarily contributed to DDR heterogeneity.

### DDR characteristics of immune cell clusters

**Figure 6:**
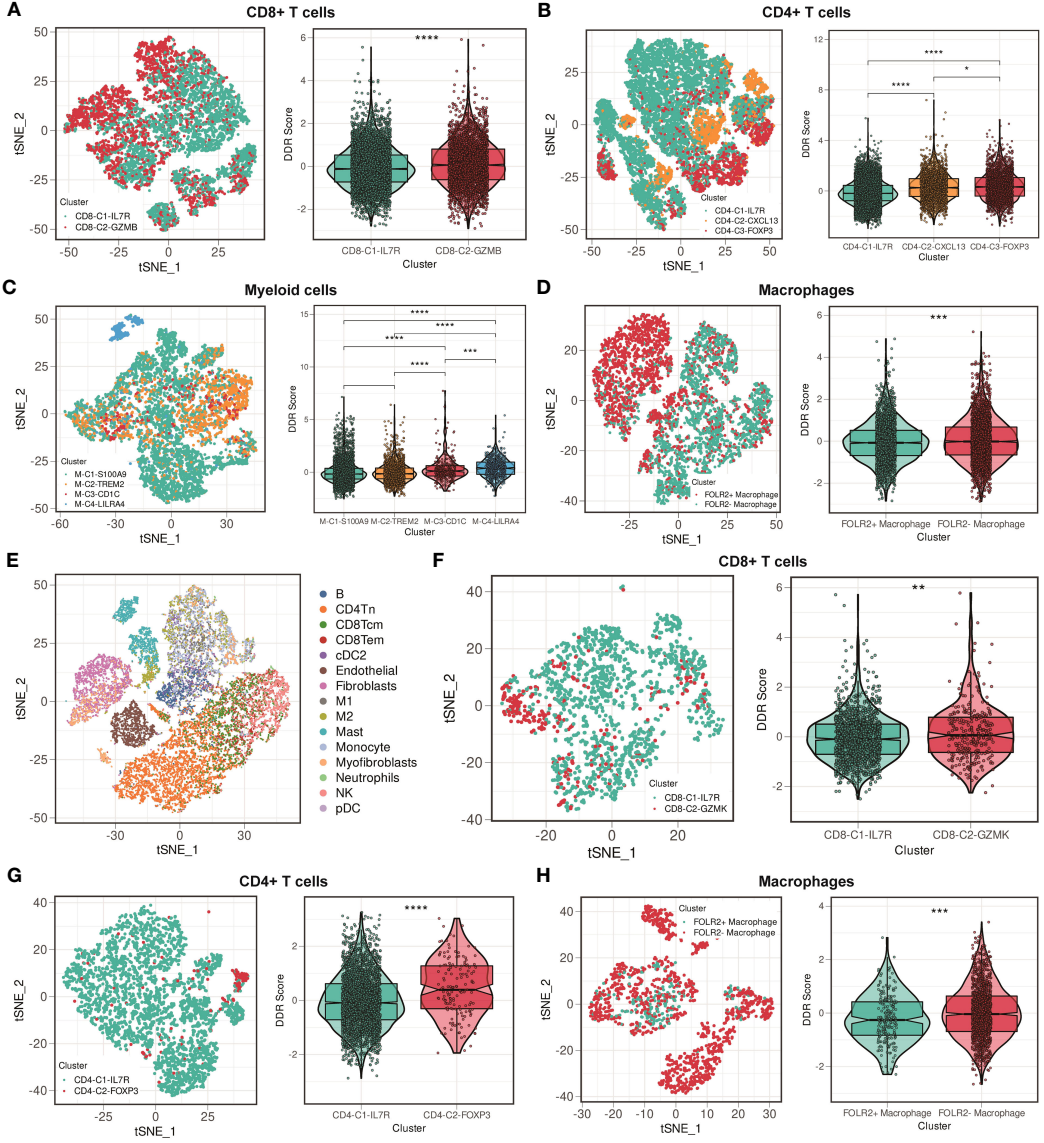
Comparisons of DDR score among immune cell clusters in the GSE176078 and GSE114727 scRNA-seq cohorts. **(A)** t-SNE plot of two clusters among CD8+ T cells, and violin plot between cluster 1 (IL7R+CD8+ T cells) and cluster 2 (GZMB+CD8+ T cells) in the GSE176078 scRNA-seq cohort. **** means *P* < 0.0001. **(B)** t-SNE plot of three clusters among CD4+ T cells, and violin plot among cluster 1 (IL7R+CD4+ T cells), cluster 2 (CXCL13+CD4+ T cells), and cluster 3 (FOXP3+CD4+ T cells) in the GSE176078 scRNA-seq cohort. **** means *P* < 0.0001 and * means *P* < 0.05. **(C)** t-SNE plot of four clusters among myeloid cells, and violin plot among cluster 1 (S100A9+ myeloid cells), cluster 2 (TREM2+ myeloid cells), cluster 3 (CD1C+ myeloid cells), and cluster 4 (LILRA4+ myeloid cells) in the GSE176078 scRNA-seq cohort. **** means *P* < 0.0001 and *** means *P* < 0.001. **(D)** t-SNE plot of two clusters among macrophages, and violin plot between cluster 1 (FOLR2+ macrophages) and cluster 2 (FOLR2- macrophages) in the GSE176078 scRNA-seq cohort. *** means *P* < 0.001. **(E)** t-SNE plot of different cell types from GSE114727 scRNA-seq cohort. **(F)** t-SNE plot of two clusters among CD8+ T cells, and violin plot between cluster 1 (IL7R+CD8+ T cells) and cluster 2 (GZMK+CD8+ T cells) in the GSE114727 scRNA-seq cohort. ** means *P* < 0.01. **(G)** t-SNE plot of two clusters among CD4+ T cells, and violin plot between cluster 1 (IL7R+CD4+ T cells) and cluster 2 (FOXP3+CD4+ T cells) in the GSE114727 scRNA-seq cohort. **** means *P* < 0.0001. **(H)** t-SNE plot of two clusters among macrophages, and violin plot between cluster 1 (FOLR2+ macrophages) and cluster 2 (FOLR2- macrophages) in the GSE114727 scRNA-seq cohort. *** means *P* < 0.001.

We further differentiated nonmalignant cells in the TME (myeloid cells, CD4+ T cells, CD8+ T cells, and macrophages) to assess their suitability for DDR-based treatment. We extracted CD8+ T cells cluster in GSE176078, and these cells were subdivided into two clusters. **Figure 6A** illustrated that cells from CD8-C1-IL7R were naive CD8+ T cells, whereas cells from CD8-C2-GZMB were cytotoxic CD8+ T cells. The result showed that the DDR score was higher in CD8-C2 cluster compared with CD8-C1 cluster (**Figure 6A**). We then classified CD4+ T cells into three clusters based on their heterogeneity: CD4-C1-IL7R, CD4-C2-CXCL13, and CD4-C3-FOXP3. The CD4-C2-CXCL13 cluster represents the main subtype of exhausted T cells, while the activation of CD4-C3-FOXP3 cluster is associated with immunosuppressive reactions. T cells from CD4-C3-FOXP3 were found to have a higher DDR score than those from CD4-C1-IL7R and CD4-C2-CXCL13 (**Figure 6B**). Following that, we detected four clusters of myeloid cells. Among these clusters, M-C1-S100A9 and M-C2-TREM2 are composed of macrophages, whereas dendritic cells comprise M-C3-CD1C and M-C4-LILRA4 (**Figure 6C**). We then compared the DDR score among four clusters. M-C4-LILRA4 exhibits high expression of LILRA4 and GZMB, indicating the presence of plasmacytoid dendritic cells (pDCs). We found that there was an obvious upregulation of DDR score in M-C4-LILRA4 compared with M-C3-CD1C (**Figure 6C**). However, M-C2-TREM2 (M2-like TAMs) displayed slightly higher levels of DDR score compared with M-C1-S100A9, a classic-type macrophage. Previous study has verified that FOLR2+ macrophages were correlated with better patient survival (39). Hence, we divided macrophages into FOLR2+ and FOLR2- macrophages based on FOLR2 expression. We found that FOLR2+ macrophages exhibited lower DDR score than FOLR2- macrophages (**Figure 6D**). Moreover, we confirmed our findings mentioned above in another scRNA-seq cohort, GSE114727. Cells were classified into 15 clusters, including B cells, mast cells, monocytes, myofibroblasts, naive CD4+ T cells, neutrophils, central memory CD8+ T cells, effector memory CD8+ T cells, classic dendritic cells, endothelial cells, fibroblasts, M1-like macrophages, M2-like macrophages, NK cells, and plasma dendritic cells (**Figure 6E**). CD8+ T cells were subdivided into two clusters, CD8-C1-IL7R (naive CD8+ T cells) and CD8-C2-GZMK (cytotoxic CD8+ T cells), and higher DDR score was observed in CD8-C2-GZMK subgroup (**Figure 6F**). Then, CD4+ T cells were classified into two clusters: CD4-C1-IL7R and CD4-C2-FOXP3 (**Figure 6G**). The result showed that CD4-C2-FOXP3 exhibited higher DDR score than CD4-C1-IL7R. Besides, we still split macrophages into FOLR2+ and FOLR2- macrophages and found that FOLR2+ macrophages had lower DDR score than FOLR2- macrophages (**Figure 6H**). Overall, these results suggest strategies for eliminating immunosuppressive immune cells that target DDR activity.

### DDR score is associated with clinical outcomes

**Figure 7:**
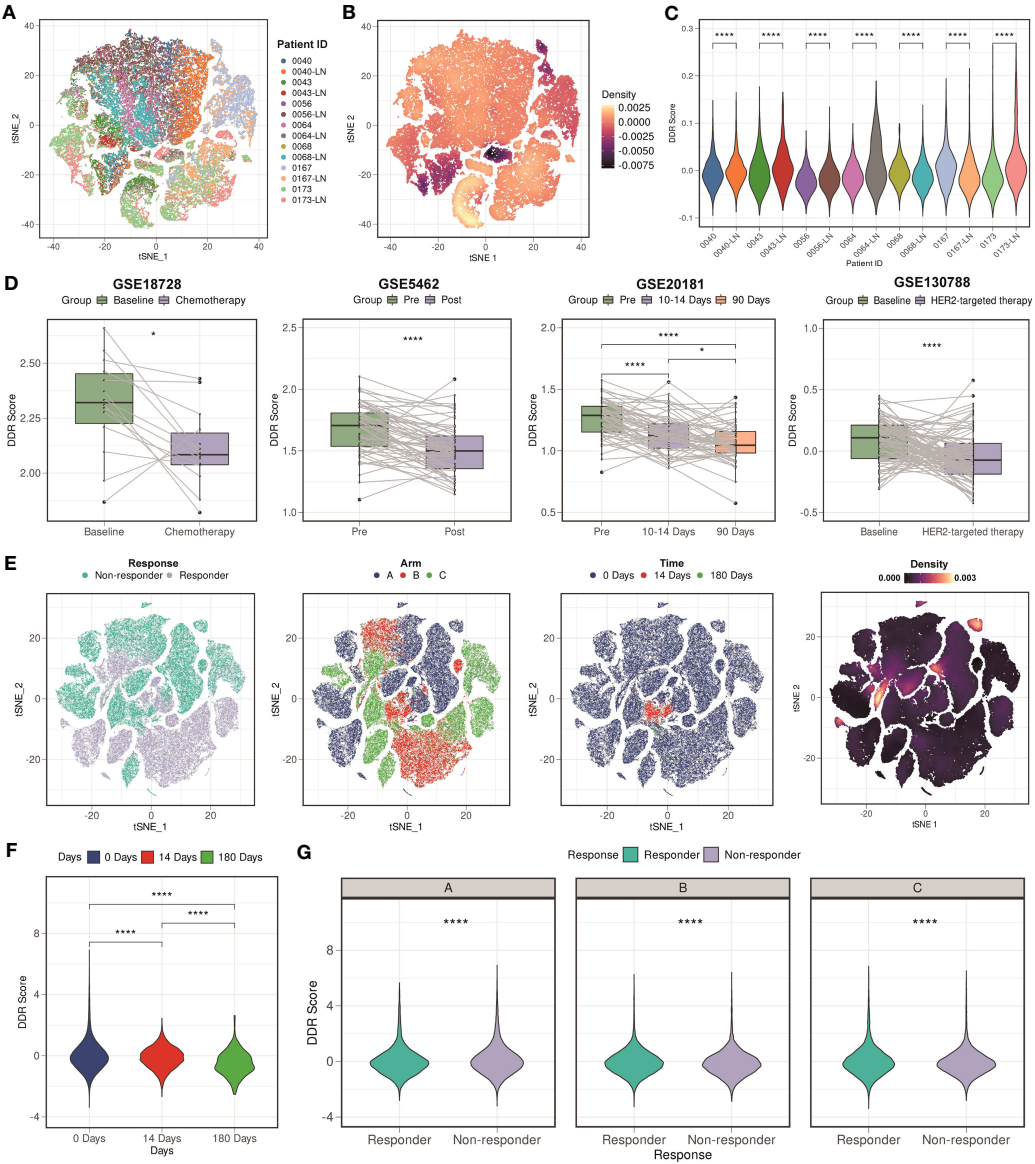
Clinical values of the DDR score. **(A)** t-SNE plot of different patient samples from GSE161529 scRNA-seq cohort. **(B)** t-SNE plot of the density of the DDR score in the GSE161529 scRNA-seq cohort. **(C)** Violin plots of the comparison among different patient samples in the GSE161529 scRNA-seq cohort. **** means *P* < 0.0001. **(D)** Boxplots of the DDR score values between different types of therapy in the GSE18728, GSE5462, GSE20181, and GSE130788 cohorts. **** means *P* < 0.0001 and * means *P* < 0.05. **(E)** t-SNE plot based on the treatment responses, different types of arms, treatment time points, and the density of the DDR score in the GSE158724 scRNA-seq cohort. **(F)** Violin plot among different treatment time points in the GSE158724 scRNA-seq cohort. **** means *P* < 0.0001. **(G)** Violin plots between different treatment responses in each arm in the GSE158724 scRNA-seq cohort. **** means *P* < 0.0001.

We then assessed whether the DDR score makes sense to the clinical outcomes. We downloaded GSE161529 scRNA-seq dataset which contained both primary and paired lymph node metastases breast cancer tissues. **Figure 7A** showed the t-distributed stochastic neighbor embedding (t-SNE) plot of different patient samples, and **Figure 7B** showed the density of the DDR score, indicating that the DDR score was mainly distributing in the lymph node metastases site compared with the primary site. We further analyzed the DDR score in each patient, and the result showed that most of the lymph node metastases sites exhibited higher DDR score compare with the paired primary sites (5 of 7 patients, **Figure 7C**). These finding indicated that the DDR score might be an indicator of the lymph node metastases.

After that, we collected three cohorts (GSE18728, GSE5462, and GSE20181) containing patients that received chemotherapy treatment. We found that the DDR score of most patients was decreasing after the treatment of chemotherapy (*P* < 0.05 in GSE18728; *P* < 0.0001 in GSE5462; *P* < 0.0001 in GSE20181, **Figure 7D**). Besides, we downloaded GSE130788 cohort containing patients that received target therapy treatment. Consistent result was found that the DDR score was lower in patients received target therapy compared with their baseline status (**Figure 7D**). Moreover, we analyzed a scRNA-seq cohort (GSE158724) which assessed the response of multiple regimens. **Figure 7E** showed the t-SNE plot based on the treatment responses, different types of arms, treatment time points, and the density of the DDR score. We verified our findings at the single-cell level, that is, the DDR score was gradually decreasing during regimens (*P* < 0.0001, **Figure 7F**). We also found that patients who were resistant to the regimens exhibited higher DDR score than those were sensitive to the regimens (*P* < 0.0001, **Figure 7G**). In summary, our findings indicated that the DDR score was strongly correlated with clinical treatment, and might be a novel indicator.

## Discussion

Despite advances in therapies, the prognosis of BC is still unfavorable due to the high recurrence rate, even after surgery. The presence of molecular heterogeneity restricts treatment options and makes survival monitoring difficult. Thus, numerous excellent studies that identified BC molecular subtypes have shed new light on BC precision medicine (3–5). Previous studies have revealed that DDR are complex biological processes, and it is well known that DDR pathways involve in the onset, progression, and therapeutic response of many diseases (6, 43). Besides, treatment strategies that target altered DDR function are gradually coming to fruition. Nonetheless, it remains unclear what role DDR plays in BC ecosystems. Large-scaled cohorts coupled with novel sequencing techniques and tools have enabled us to explore the DDR complexity.

Our study found that, among BC patients, there were two subtypes with distinct clinical and molecular characteristics: the DDR-suppressed subtype and the DDR-active subtype. A superior survival rate is found for tumors in the DDR-suppressed class, while those with the DDR-activated class tend to have inferior prognoses and clinically aggressive behavior. Genomic and transcriptomic variations were found between two subgroups, suggesting that the DDR heterogeneity should be incorporated into personalized therapy development. Furthermore, we constructed the DDR score and scRNA-seq analyses revealed that the DDR heterogeneity of BC was primarily caused by malignant cells. We also found that the TME was also crucial to DDR plasticity. Moreover, we found that, at the single-cell level, the DDR score can be differentiated between immune and stromal cell subtypes. Besides, it appeared that as a part of the DDR process, immune-suppressive cells like FOXP3+ CD4+ T cells were involved. It is anticipated that our findings might aid the understanding of the DDR heterogeneity within BC as well as gain a new insight into how to tailor therapies accordingly.

Despite its advantages, our study has some limitations as well. First, multiple expression detection platforms were used, and large-scaled studies are necessary to verify the robustness of the clusters. Second, data from multi-cohorts were primarily used to assess DDR-related survival and molecular features, and more information about DDR subtype alterations might be provided by future *in vitro* and *in vivo* investigations. Third, dataset like METABRIC only contains targeted panel sequencing, and the majority of the samples might do not have sufficient mutational load to perform signature analysis. Fourth, other novel technologies such as proteomics and spatial transcriptomes were not enrolled to analyze in this study.

## Conclusion

Consequently, this study offers a novel perspective for understanding the DDR heterogeneity of BC. Identifying specific DDR subtype characteristics helps BC patients to make informed clinical decisions. The robust DDR clusters demonstrated that DDR-related pathways were vital to clinical outcomes and indicated potential target during DDR activation. With the advent of scRNA-seq technique, we are better equipped to understand the complexity of DDR in BC. In the future, novel technologies such as proteomics and spatial transcriptomes will add to the ability to characterize DDR status.

## Data availability statement

The datasets presented in this study can be found in online repositories. The names of the repository/repositories and accession number(s) can be found in the article/**Supplementary Material**.

## Author contributions

PL: Data curation, Formal Analysis, Writing – original draft. XD: Data curation, Formal Analysis, Writing – original draft. HZ: Data curation, Formal Analysis, Writing – original draft. JX: Formal Analysis, Visualization, Writing – original draft. YK: Funding acquisition, Investigation, Writing – original draft. YZ: Project administration, Supervision, Writing – review & editing. AY: Project administration, Supervision, Writing – review & editing. XL: Project administration, Supervision, Writing – review & editing.
