# Supplementary material for: Multi-omics analyses unravel DNA damage repair-related clusters in breast cancer with experimental validation
Source: Front Immunol. 2023 Oct 31;14:1297180. doi: 10.3389/fimmu.2023.1297180 (PMC10644223; doi:10.3389/fimmu.2023.1297180)
Supplement: Supplementary file 1 [file DataSheet_1.pdf]

Supplementary Figure S1. Boxplots of the PRAME expression among different breast cancer subtypes. ** means P < 0.0001.**

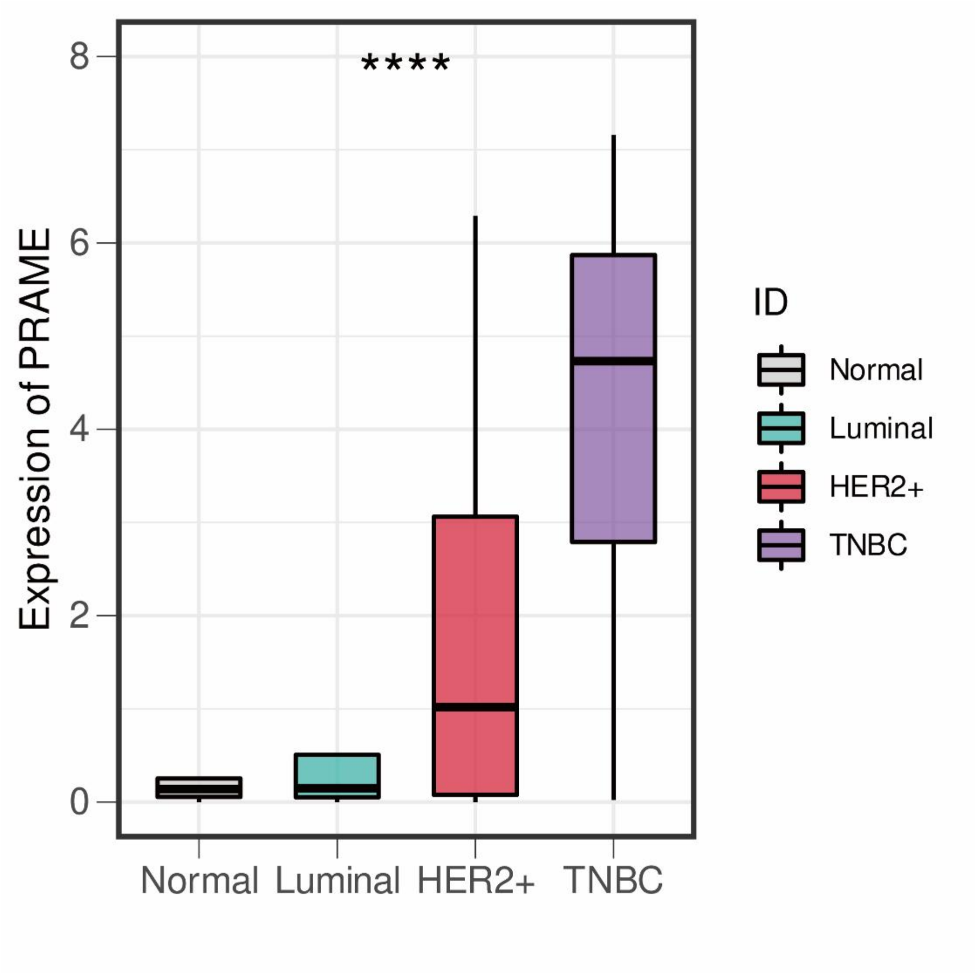

Supplementary Table S1. 276 DDR-related genes

Base Excision Repair (BER)	Nucleotide Excision Repair (NER)	Mismatch Repair (MMR)	Fanconi Anemia (FA)	Homology-dependent recombination (HDR)	Non-homologous End Joining (NHEJ)	Direct Repair (DR)	Translesion Synthesis (TLS)	Nucleotide pools (NP)	Damage Sensor etc.
APLF	LIG1	LIG1	XRCC2	LIG1	DNTT	ASCC3	POLB	NUDT1	ATM
APTX	PCNA	EXO1	APITD1	MRE11A	LIG4	ALKBH2	POLM	NUDT15	ATR
LIG1	POLD1	HMGB1	BARD1	NBN	MRE11A	ALKBH3	UBE2A	NUDT18	ATRIP
LIG3	POLD2	MLH1	BLM	PARG	NBN	MGMT	PCNA	RRM1	ATRX
PARG	POLD3	MLH3	BRCA1	PARP1	NHEJ1		HLTF	RRM2	CHAF1A
PARP1	POLD4	MSH2	BRCA2	PARPBP	PARG		MAD2L2		CHEK1
PARP3	RFC1	MSH3	BRE	RAD50	PARP1		POLH		CHEK2
PNKP	RFC2	MSH6	BRIP1	TP53BP1	PARP3		POLI		CLK2
POLB	RFC3	PCNA	ERCC1	XRCC2	PNKP		POLK		DCLRE1A
POLL	RFC4	PMS1	ERCC4	XRCC3	POLB		POLN		DCLRE1B
XRCC1	RFC5	PMS2	FAAP100	EXO1	POLL		POLQ		DUT
HMGB1	RPA1	POLD1	FAAP24	PCNA	POLM		RAD18		GADD45A
PCNA	RPA2	POLD2	FAAP20	POLD1	PRKDC		REV1		GADD45G
POLD1	RPA3	POLD3	FAN1	POLD2	RAD50		REV3L		HUS1
POLD2	RPA4	POLD4	FANCA	POLD3	RNF168		SHPRH		MDC1
POLD3	CCNH	RFC1	FANCB	POLD4	RNF8		UBE2B		MPLKIP
POLD4	CDK7	RFC2	FANCC	RFC1	TP53BP1		UBE2N		MRPL40
RFC1	CETN2	RFC3	FANCD2	RFC2	XRCC4		UBE2V2		NABP2
RFC2	CUL3	RFC4	FANCE	RFC3	XRCC5		USP1		PER1
RFC3	CUL4A	RFC5	FANCF	RFC4	XRCC6		WDR48		POLA1
RFC4	CUL5	RPA1	FANCG	RFC5	DCLRE1C				POLG
RFC5	DDB1	RPA2	FANCI	RPA1	FAM175A				PRPF19
ALKBH1	DDB2	RPA3	FANCL	RPA2	RIF1				RAD1
APEX1	ERCC1	RPA4	FANCM	RPA3					RAD17
APEX2	ERCC2		HELQ	RPA4					RAD9A
FEN1	ERCC3		HES1	BARD1					RIF1
HMGB2	ERCC4		MAD2L2	BLM					RNMT
MBD4	ERCC5		PALB2	BRCA1					RRM2B
MPG	ERCC6		RAD51	BRCA2					SETMAR
MUTYH	ERCC8		RAD51C	BRIP1					SLX4
NEIL1	GTF2H1		RMI1	DMC1					TOPBP1
NEIL2	GTF2H2		RMI2	DNA2					TP53
NEIL3	GTF2H3		SLX1A	EID3					TREX1
NTHL1	GTF2H4		SLX4	EME1					TREX2
OGG1	GTF2H5		STRA13	EME2					TYMS
PARP2	MMS19		TELO2	ERCC1					PTEN
PARP4	MNAT1		TOP3A	FANCM					TDP2
POLE	POLE		TOP3B	FEN1					ENDOV
POLE2	POLE2		UBE2T	GEN1					SPRTN
POLE3	POLE3		USP1	H2AFX					RNF4
POLE4	POLE4		WDR48	HELQ					SMARCA4
POLK	RAD23A			HFM1					IDH1
SMUG1	RAD23B			INO80					SOX4
TDG	RBX1			KAT5					WEE1
TDP1	TCEA1			MUS81					RAD9B
UNG	TCEB1			NFATC2IP					AEN
WRN	TCEB2			NSMCE1					PLK3
	UVSSA			NSMCE2					EXO5
	XAB2			NSMCE3					CDC5L
	XPA			NSMCE4A					BCAS2
	XPC			PALB2					PLRG1
				PARP2					YWHAB
				PAXIP1					YWHAG
				POLH					YWHAE
				POLQ					CDC25A
				PPP4C					CDC25B
				PPP4R1					CDC25C
				PPP4R2					BABAM1
				PPP4R4					BRCC3
				RAD51					TTK
				RAD51B					SMARCC1
				RAD51C					SWI5
				RAD51D					MORF4L1
				RAD52					RNF169
				RAD54B					HERC2
				RAD54L					
				RBBP8					
				RDM1					
				RECQL					
				RECQL4					
				RECQL5					
				RMI1					
				RMI2					
				RTEL1					
				SHFM1					
				SLX1A					
				SLX1B					
				SLX4					
				SMARCAD1					
				SMC5					
				SMC6					
				SPO11					
				SWSAP1					
				TOP3A					
				TOP3B					
				UIMC1					

				WRN					
				ZSWIM7					

Supplementary Table S2. Primers for qRT-PCR detection

PRAME	Forward	CAGGACTTCTGGACTGTATGGT
	Reverse	CTACGAGCACCTCTACTGGAA
β-actin	Forward	CATGTACGTTGCTATCCAGGC
	Reverse	CTCCTTAATGTCACGCACGAT
